# FGF23 level in poultry chicken, a systematic review and meta-analysis

**DOI:** 10.3389/fphys.2023.1279204

**Published:** 2023-10-16

**Authors:** Hossein Poorhemati, Melody Ghaly, Gulzhakhan Sadvakassova, Svetlana V. Komarova

**Affiliations:** ^1^ Department of Biological and Biomedical Engineering, McGill University, Montreal, QC, Canada; ^2^ Shriners Hospitals for Children–Canada, Montreal, QC, Canada; ^3^ Faculty of Dental Medicine and Oral Health Sciences, McGill University, Montreal, QC, Canada

**Keywords:** phosphate, calcium, FGF23, vitamin D, broiler, egg-laying hen, diet

## Abstract

**Introduction:** In vertebrates fibroblast growth factor 23 (FGF23) is a phosphate regulating hormone closely linked to calcium regulation by vitamin D and parathyroid hormone (PTH). Although phosphorus, calcium and vitamin D are important for poultry well-being, relatively little is known about their levels of FGF23. Our objective was to quantitatively estimate the blood FGF23 level in birds, and to examine its relationship to diet and blood levels of other components of phosphate and calcium homeostasis.

**Methods:** A systematic search of Agricola, Embase and Medline identified 86 studies focused on FGF23 in birds, from which 12 manuscripts reporting data for 60 independent groups of chickens were included in the analysis.

**Results:** FGF23 levels were 256 pg/ml (Confidence interval (CI): 215, 297) in broilers (39 datasets containing 435 birds), and 256 pg/ml (CI: 178, 339) in egg-laying hens (21 datasets containing 208 birds). FGF23 levels did not correlate with dietary phosphorus, calcium or vitamin D, or with plasma calcium or PTH. FGF23 levels demonstrated a trend to positively correlate with plasma phosphate and a strongly and positive correlation with plasma vitamin D.

**Discussion:** This study provides normative estimates of FGF23 levels in poultry birds and new insights into the regulation of calcium and phosphate homeostasis.

## Introduction

Fibroblast growth factor 23 (FGF23), vitamin D, and parathyroid hormone (PTH) are hormones regulating blood phosphate and calcium homeostasis by affecting intestine, kidney, and bone (Renkema et al., 2008; Arnold et al., 2021). While the function of PTH and vitamin D is well-studied in mammals (Dittmer and Thompson, 2011; Stenhouse et al., 2022) and birds (Dacke et al., 2015), the critical role of FGF23 in phosphate homeostasis was identified relatively recently (Martin et al., 2012). FGF23 produced in bone by osteocytes (Agoro et al., 2020) decreases renal phosphate reabsorption and consequently increases urinary phosphate excretion. In addition, FGF23 decreases the availability of active vitamin D by blocking the expression of kidney 1-α-hydroxylase, the enzyme converting inactive form of vitamin D (25(OH)D) to the active form (1,25(OH)_2_D), and by increasing the expression of 24-hydroxylase which degrades 1,25(OH)_2_D (Shimada et al., 2004). Reduction of active vitamin D results in decrease of the intestinal absorption of phosphate. In mammals, FGF23 role is now well-established (Shimada et al., 2004), and in birds the levels and regulation of FGF23 was addressed in numerous studies making possible a comprehensive analysis of published literature.

The importance of calcium and phosphorous was long recognized by poultry science researchers. In egg-laying hens, a considerable volume of calcium is needed for the eggshell formation, while in broilers there is a more than normal requirement of calcium and phosphate to provide the bird with the bones that can bear their rapidly increasing body mass (Bar, 2008; Fleming, 2008). Low level of dietary phosphate can result in abnormal bone mineralization and increase the bird’s fatality (Proszkowiec-Weglarz and Angel, 2013; Wei et al., 2022), while high concentration of phosphate in the poultry feed leads to increased operational and environmental costs (Nahm, 2007; Andretta et al., 2021). Therefore, the effect of diet on the blood level of phosphate has been studied (Broz et al., 1994; Pelicia et al., 2009; Jing et al., 2018; Li et al., 2020) to determine the optimum phosphate concentration that reduces the related costs and at the same time ensures the welfare of the birds. Better understanding of basic relationships between blood levels of calcium, phosphorous, and their hormonal regulators would benefit optimization of multiple dietary parameters against multiple homeostatic targets.

Given a potential importance of FGF23 in phosphate homeostasis and its relevant novelty, our overall goal was to examine the relationships between FGF23 and other regulators of calcium and phosphate homeostasis in birds. We performed the systematic review and meta-analysis of studies that measured the blood level of FGF23 in poultry birds with the primary objective of providing a quantitative estimate of FGF23 levels in birds and the secondary objective of investigating possible correlations between blood FGF23 levels and dietary and blood levels of other players in calcium and phosphate homeostasis.

## Methods

**Study selection:** A systematic search strategy combining terms describing FGF23 with terms describing different types of birds was constructed (Supplemental Material). The search of three databases: Agricola, Embase, and Medline was performed. The title/abstracts were screened by two reviewers (MG and GS for the original screen, HP and SVK for the update) for mentioning FGF23 in any type of bird. Full text screening was performed by MG and HP for reporting of quantitative values of FGF23 in untreated birds. In all cases, the reviewers worked independently.

**Data extraction:** For all studies included in the review the following items were extracted: Authors and year, diet calcium (g/kg), diet phosphorous (g/kg), diet vitamin D (IU/kg), age (days), sex, in the case of poultry, breed and breed type (broiler or egg-laying hen), blood calcium (mmol/L), blood phosphate (mmol/L), blood vitamin D (pg/mL), blood PTH (pg/mL), blood FGF23 (pg/mL), FGF23 measurement method. For all measurements we extracted mean, measure of variance (SEM or SD) and sample size. When the study did not report all the items listed above, it was excluded from subgroup analysis for the missing item. When the range of samples rather that exact sample size was reported, we extracted the lower number of the range to provide a more conservative estimate. When data were presented in graphs rather than tables, WebPlotDigitizer was used to extract the values.

**Data preparation:** The absolute value of the blood FGF23 from untreated samples were used as the study-level outcome. While most studies reported SD or SEM as their measure of variance, we noticed that in several cases the reported standard errors were the same for several datasets extracted from that study (Jiang et al., 2015; Ren et al., 2017a; Ren et al. 2017b; Ren et al. 2019). To avoid unrealistic weight given to these datasets in the variance based meta-analysis, we introduced a more conservative estimate for the standard deviation, that combined the reported standard deviation (or calculated from the reported standard error) for the dataset *i*, ${SD}_{i,Reported}$ , and the standard deviation calculated for all the reported datasets obtained from study *j* that had similar age as dataset *i*:

$${SD}_{i,combined}=\sqrt{{SD}_{i,Reported}^{2}+{SD}_{Age,Calculated}^{2}}$$ (1)

With the improved standard deviation, we recalculated the standard error using ${SE}_{i,combined}=\frac{{SD}_{i,combined}}{\sqrt{n_{i}}}$ where $n_{i}$ is the sample size for dataset *i*.

**Meta-analytic model:** We used the Robust Bayesian Model-Averaged meta-analysis (RoBMA) introduced by Maier et al. (Maier et al., 2020) using the RoBMA package in JASP. RoBMA allows to use Bayesian statistics to assess relative probability of 12 different meta-analytic models based on three characteristics: a) whether there is an effect (null or alternative hypothesis), b) whether there is homogeneity (fixed effect or random effects), and c) whether the publication bias is present, marginally present, or absent. This approach allows to estimate the overall effect with the confidence interval as well as the probability of reporting bias.

**Additional analysis:** To assess the effect of age and breed, subgroup analysis was performed and the significance of the difference between the subgroup was examined using Bayesian ANOVA. To investigate the correlation between blood FGF23 levels and other parameters, we used Bayesian correlation, in which Pearson’s rho was calculated as the population correlation coefficient and the BF_10_ was calculated for the alternative hypothesis chosen as *correlated*. All analyses were performed in JASP.

**Outcome reporting:** We report effect size (ES) as raw value of blood FGF23 with lower and upper limits of 95% CI calculated by RoBMA as: ES [Lower CI, Upper CI]. In accordance with convention of Bayesian statistics (Quintana and Williams, 2018), the statistical evidence was considered moderate when BF_10_ > 3, strong when BF_10_ > 10, very strong when BF_10_ > 30, and extreme when BF_10_ > 100.

**Software:** Endnote X7 and Rayyan were used for reference management. WebPlotDigitizer was in part used for data extraction. Microsoft Excel 365 (version 2102) was used for data management and initial calculations. JASP (version 0.14.1) was used for meta-analysis. Microsoft Excel 365 (version 2102) and CorelDRAW 2017 (version 19.1.0.419) were used to prepare the figures.

## Results

### Identifying studies reporting FGF23 levels in birds

**FIGURE 1:**
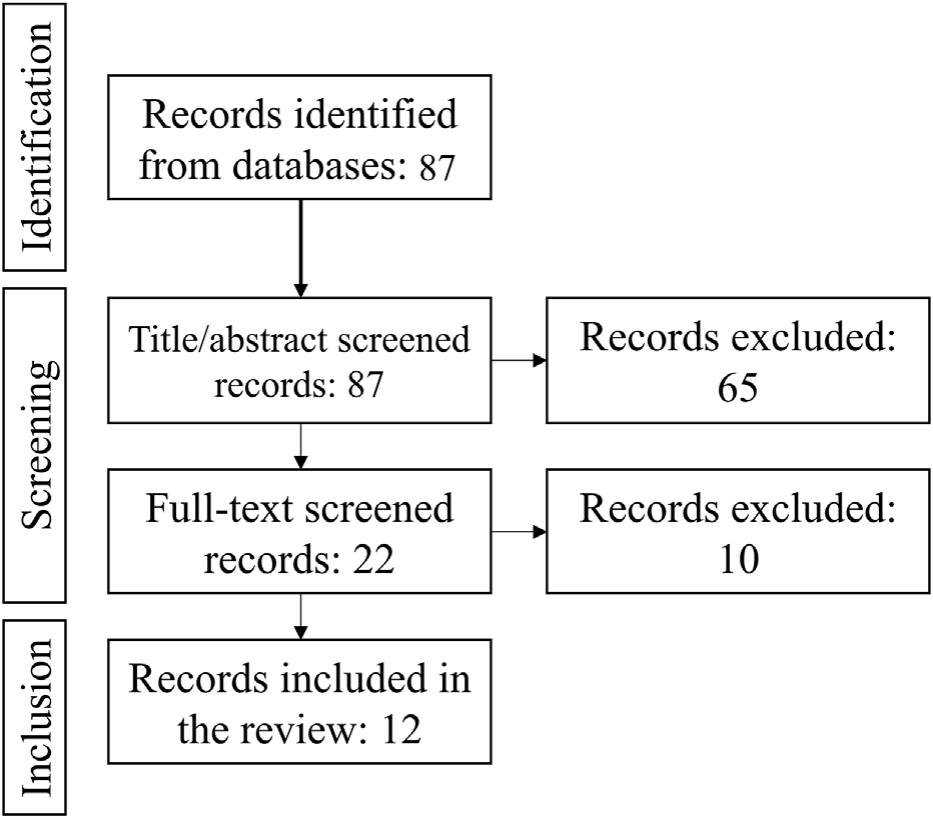
Preferred Reporting Items for Systematic reviews and Meta-Analyses (PRISMA) diagram for the information flow in the study, the number of studies at each stage of screening is indicated.

A systematic search of studies that reported blood levels of FGF23 in birds in Agricola, Embase and Medline identified 86 unique studies. After title/abstract screening, 22 studies remained, 10 of which were excluded after full text screening. Thus, 12 studies reported the blood FGF23 level in untreated birds (Figure 1). In all selected papers, the birds under study were poultry chicken, with three papers describing broilers, and nine describing egg-laying hens (Table 1). All papers were checked for the quality of reporting of the study population and methods. From the context of our study focused on the regulator of phosphate homeostasis closely linked with calcium homeostasis, we may foresee differences between broilers selected to gain weight in a short time for the purpose of meat production, and egg-laying hens selected for egg productions that requires movement of large volumes of calcium (Bar, 2008). Therefore, we conducted the meta-analysis in broilers and egg-laying hens separetely.

**TABLE 1:** Characteristics of included studies. For each study, type, age, sex, and breed of poultry chickens are indicated. The ID of independent datasets used in the manuscript are in the last column.

Author	Age (days)	Sex	Type	Breed	Dataset ID
Jiang et al., 2015	21,42,63	M	Broiler	Chinese yellow-feathered	S1-S24
Horvat-Gordon et al., 2019	21	F/M	Broiler	Cobb X Cobb	S25-S26
Cao et al., 2021	14,28,42	M	Broiler	Arbor Acres male broiler	S27-S39
Ren et al., 2017a	7,14	F/M	Egg-laying hen	Single Comb White Leghorn	S40-S45
Ren et al., 2017b	203	F	Egg-laying hen	Single Comb White Leghorn	S46
Ren et al., 2017a	14	F/M	Egg-laying hen	Single Comb White Leghorn	S47-S48
Ren et al., 2019	245	F	Egg-laying hen	Hy-Line Brown	S49
Ren et al., 2019	14,455	F/M	Egg-laying hen	Single Comb White Leghorn	S50-S52
Ren et al., 2019	511	F	Egg-laying hen	Single Comb White Leghorn	S53
Ren et al., 2020 ^a^	280	F	Egg-laying hen	Hy-Line Brown	S54-S55
Ren et al., 2020 ^a^	280	F	Egg-laying hens	Hy-Line Brown	S56
Sadvakassova et al., 2022	56,112	F	Egg-laying hen	Lohmann Brown-Lite	S57-S60
				Lohmann LSL-Lite	

^a^ These studies were conducted by the same team in the same year and the birds are of the same breed and age. However, careful comparison of the data suggests that different chickens from the same flock were used in these studies, and the data were treated as independent samples.

**FIGURE 2:**
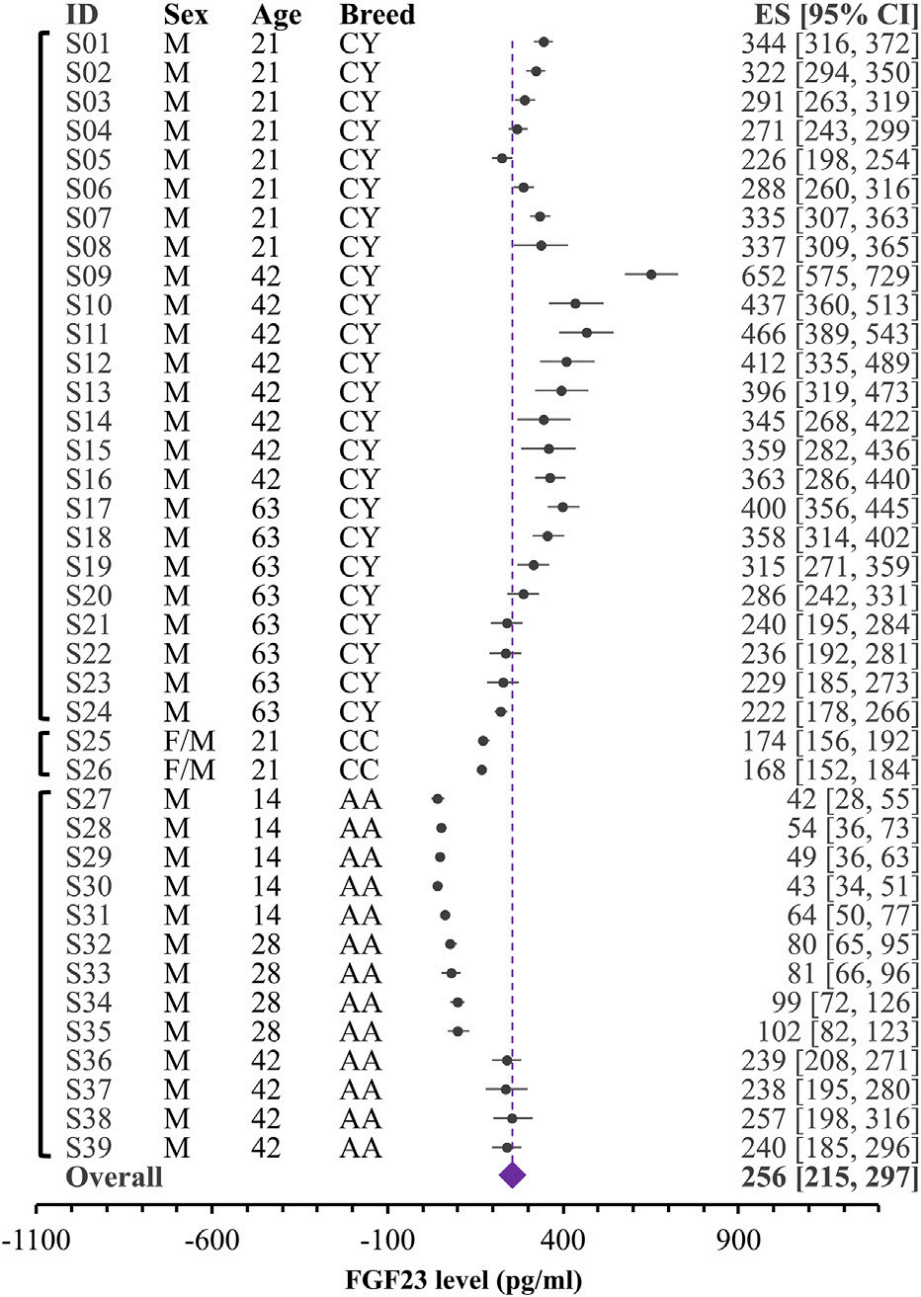
Forest plot for blood levels of the FGF23 (pg/mL) in broilers. Black circles and lines represent the single dataset effect size and corresponding 95% CI. The diamond represents the overall effect size with 95% CI. Vertical brackets on the left indicate datasets coming from the same study. Indicated are breeds (CY: Chinese yellow-feathered broilers; CC: Cobb Cobb chicks; AA: Arbor Acres male broilers), ages and sex for each group.

**FIGURE 3:**
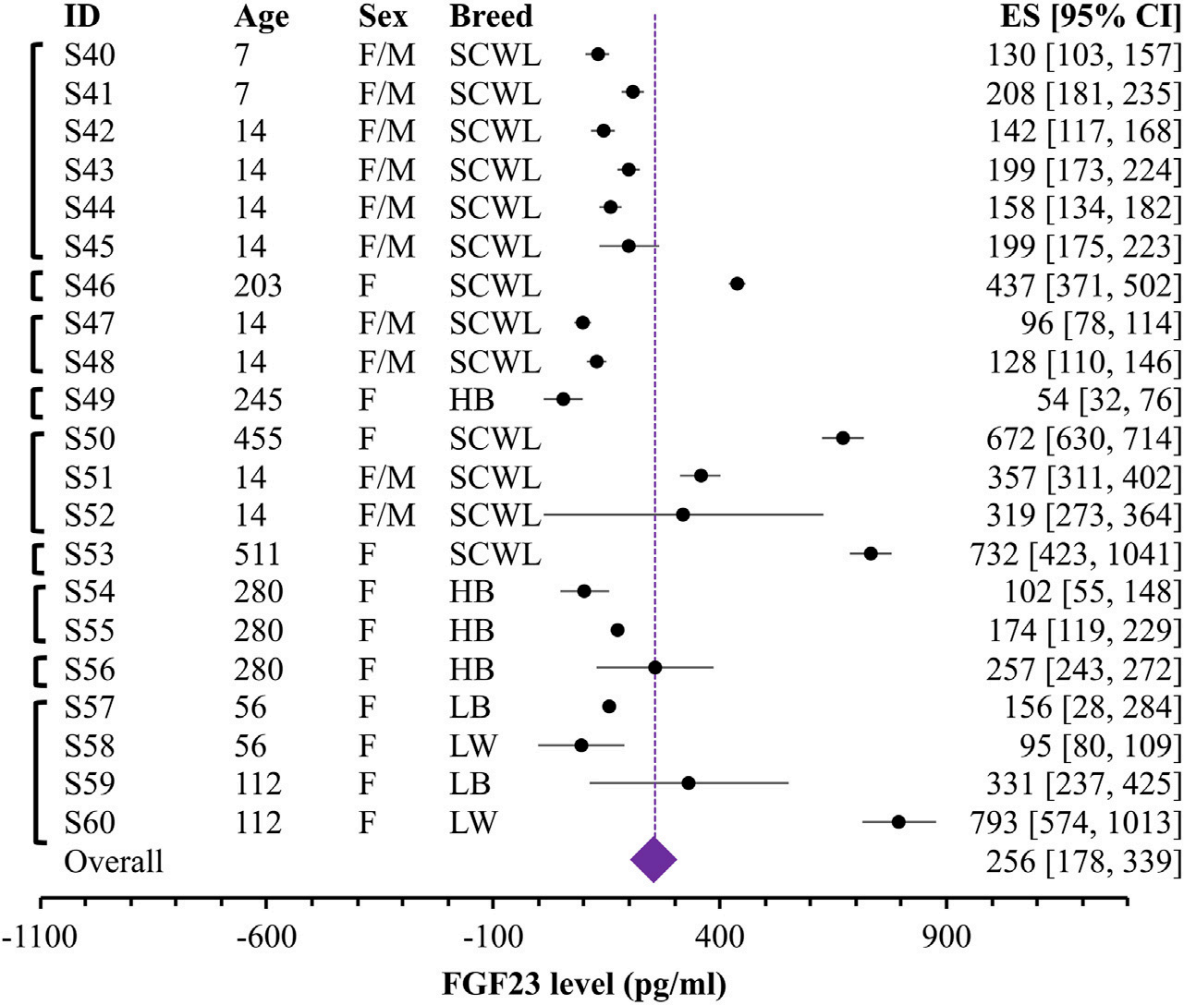
Forest plot for blood levels of the FGF23 (pg/mL) in egg-laying chicken. Black circles and lines represent the single dataset effect size and corresponding 95% CI. The diamond represents the overall effect size with 95% CI. Vertical brackets on the left indicate datasets coming from the same study. Indicated are breeds (SCWL: Single Comb White Leghorn; HB: Hy-Line Brown laying hens; LB: Lohmann Brown-Lite; LW: Lohmann LSL-Lite), ages and sex for each group.

During data extraction, if the study reported measurements in birds of different breed, diet, or age, they were extracted as separate datasets. If the measurements were performed on the same birds in different times during a short period of time (less than a week), we averaged the values of all time points and used them as one dataset. We noticed several cases of unclear reporting of variances, where the reported measure was indicated to be a standard error, but it was the same small value for many datasets in that study. To avoid unrealistic weight given to these datasets in the variance based meta-analysis, we combined the reported variance measure for the dataset with the standard deviation calculated for all the reported datasets obtained from that study. In total, we extracted 61 datasets, containing data from 643 individual chickens combined in 60 independent groups of chickens from 7 breeds, of which 39 datasets were in 14–63 days old broilers and 21 datasets in 7–511 days old egg-laying hens. Table 1 presents the study characteristics and corresponding dataset IDs that are listed in Figure 2 and Figure 3.

### Quantitative estimates of blood FGF23 levels in poultry birds

Given unclear reporting of variance, we decided to use Bayesian meta-analysis, which better accommodates the uncertainties in the data. We used a recently developed Robust Bayesian Meta-Analysis (RoBMA) (Maier et al., 2020), which allows to estimate posterior probabilities for 1) the presence of an effect; 2) the degree of heterogeneity (fixed effect vs random effects scenario) and 3) evidence of publication bias. While in our case, the presence of effect is irrelevant, because we summarize raw values rather than looking for the effect of intervention, degree of heterogeneity and evidence of bias are difficult to assess within a standard meta-analysis that strongly relies on the proper reporting of variance. We combined all the datasets studying blood FGF23 level in broilers (Figure 2) and egg-laying hens (Figure 3). The overall estimate for FGF23 was identical in broilers, 256 pg/mL with the 95% CI of [215, 297] (n = 39 datasets reporting N = 435 samples), and in egg-laying hens, 256 pg/mL [178, 339] (n = 21 datasets reporting N = 208 samples). RoBMA analysis indicated strong evidence for the random effects scenario (Bayes factor of infinite and 3.431e +243, for broilers and egg-laying hens, respectively). RoBMA analysis indicated absence of evidence for publication bias (Bayes inclusion factor of 1.005 and 1.541 for broilers and egg-laying hens, respectively). Model overview table for each case is presented at the Supplemental Table S1, S2.

### Effects of covariates on FGF23 levels in poultry birds

**FIGURE 4:**
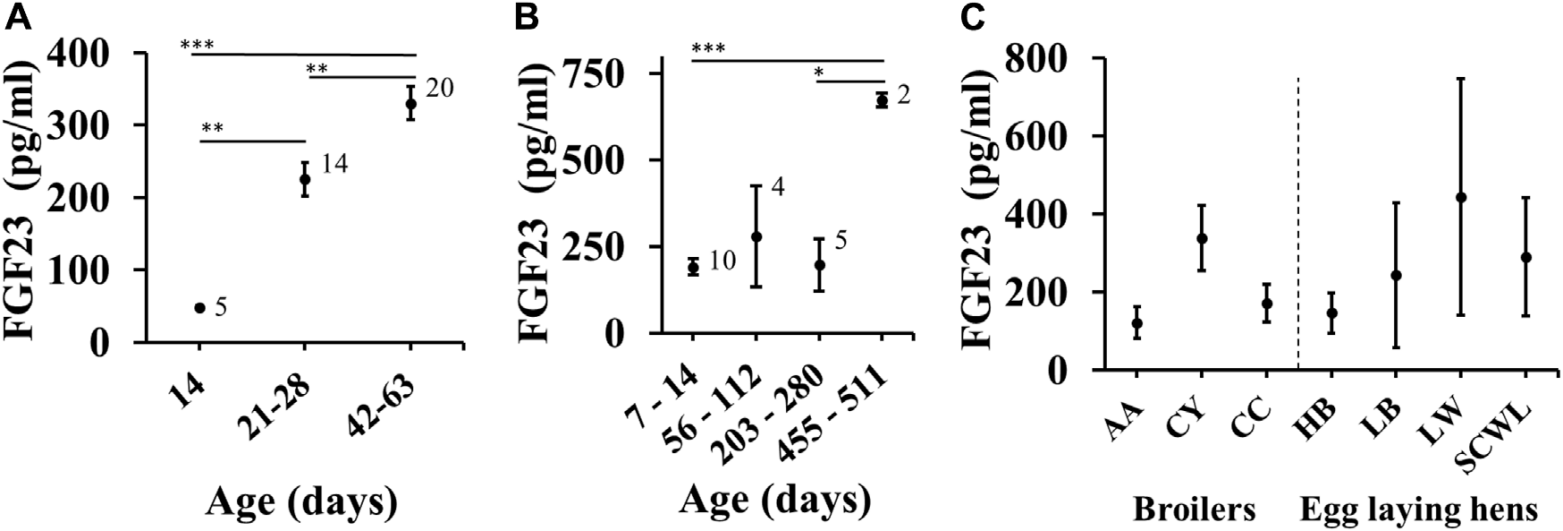
Effect of age and breed on FGF23 level. **(A, B)** Subgroup analysis for FGF23 levels in chicken of indicated ages in broilers **(A)** and egg laying hens **(B)**. Age categories were selected to maximize the chance of each subgroup containing datasets from different studies. **(C)** Subgroup analysis for egg-laying hens of 14–280 days in age separated by the breed type as brown (Hy-Line Brown, Lohmann Brown-Lite) and white (Single Comb White Leghorn, Lohmann LSL-Lite). The number on top indicates the number of datasets included in each subgroup. Asterisks indicate significance assessed by Bayesian ANOVA with Tukey post-test (*BF_10_ > 10; ***BF_10_ > 100).

Next, we examined the effects of covariates on FGF23 levels in broilers and egg laying hens. To assess the effect of age, we used subgroup analysis rather than meta-regression to minimize the potential influences of individual studies by selecting age categories that maximize the chance of each subgroup containing datasets from different studies. Both in broilers (Figure 4A) and in egg-laying hens (Figure 4B), aging was associated with significant increase in blood FGF23 level. It was not possible to assess sex differences, since the male and female chicken were from different poultry categories, and even when mixed sexes were used in young chicks (F/M status in several datasets), the data was not separated by sex. Finally, the blood FGF23 levels were similar in birds of different breeds (Figure 4C).

### Correlations between FGF23 and other players in calcium and phosphate homeostasis

**FIGURE 5:**
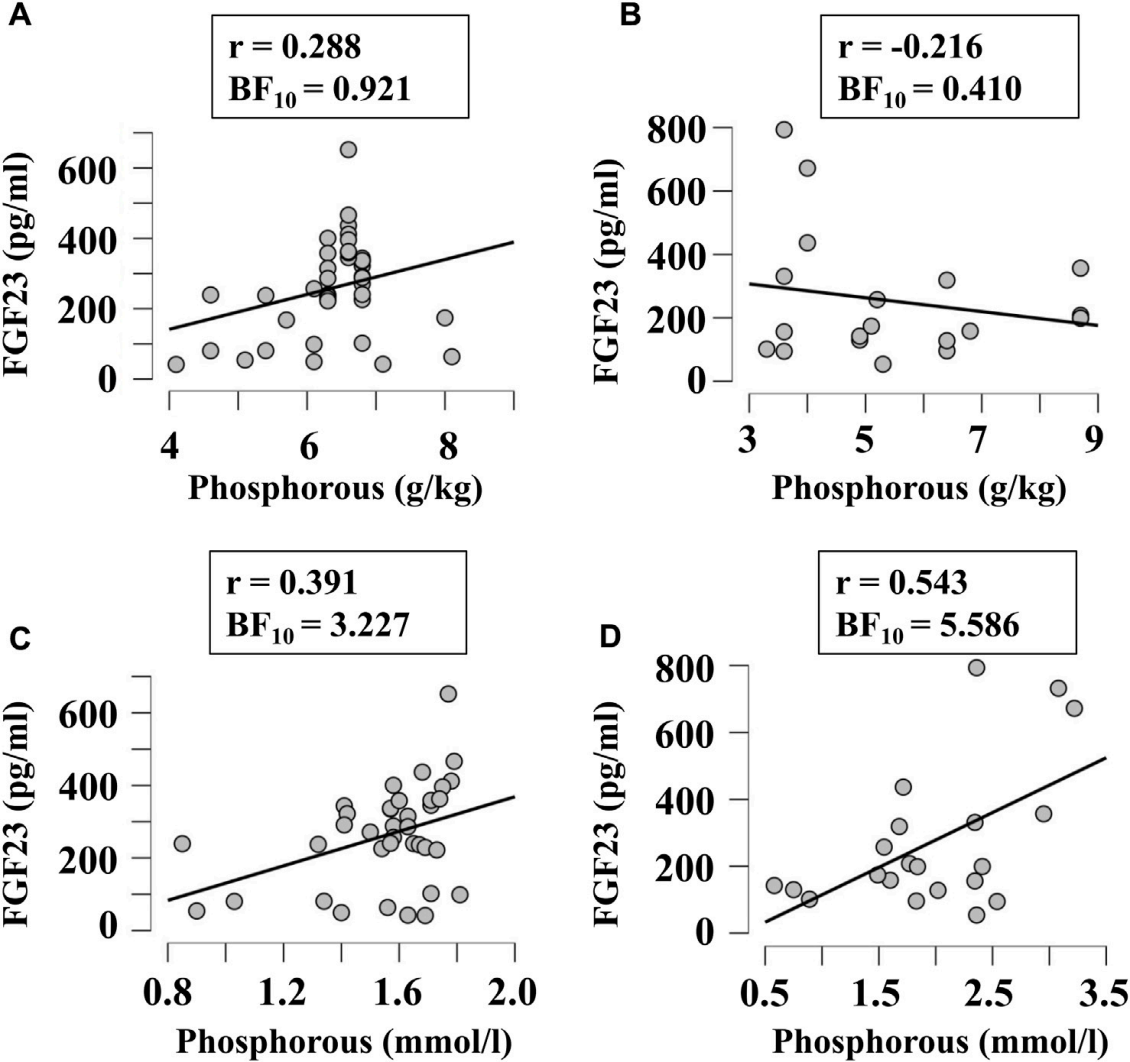
Correlation of blood FGF23 levels with dietary **(A, B)** and plasma **(C, D)** concentrations of phosphorus in broilers **(A, C)** and egg-laying hens **(B, D)**. The regression was studied using Bayesian correlation. The number of datasets, Person’s r and Bayes factor are reported below each plot.

**FIGURE 6:**
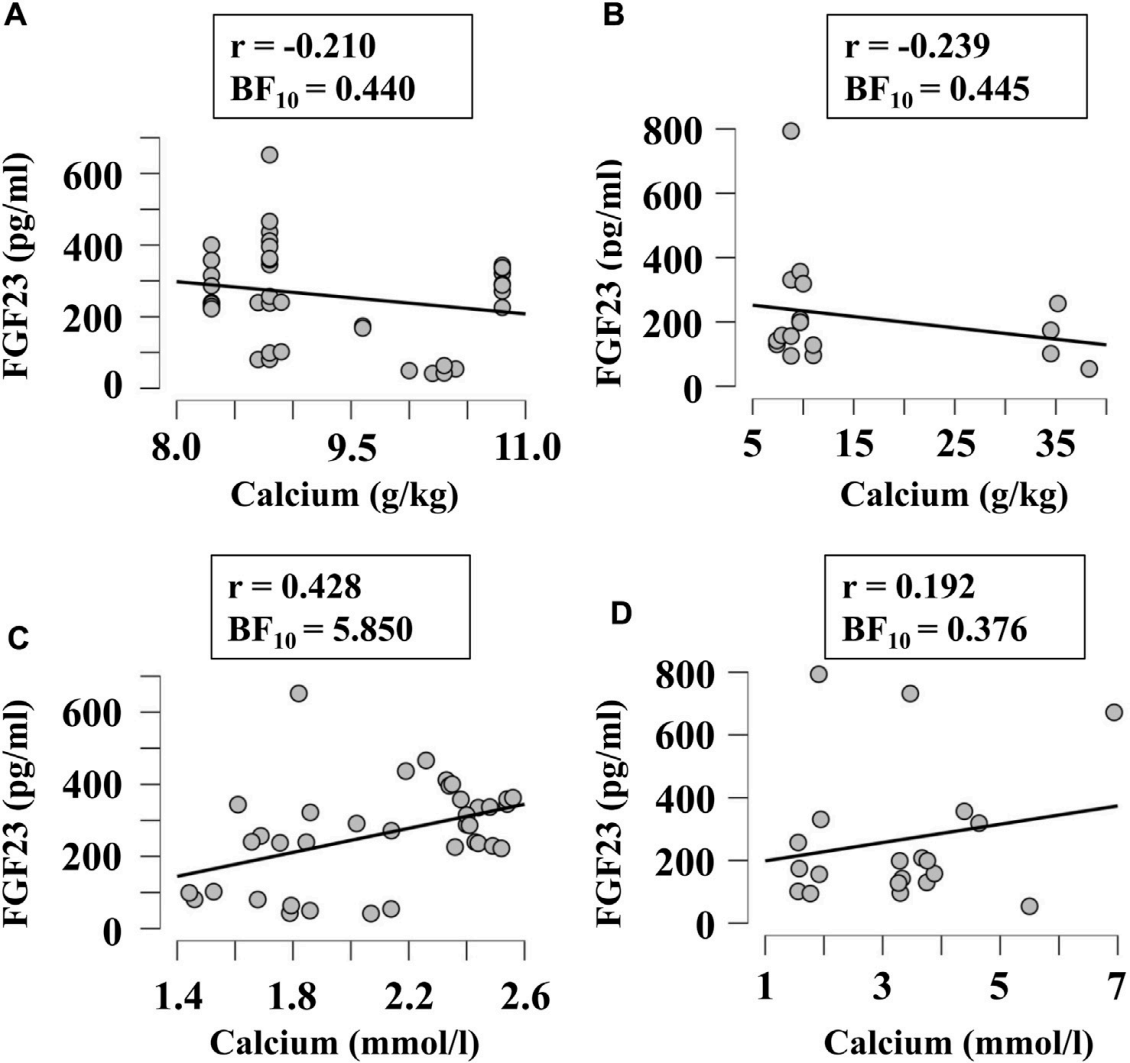
Correlation of blood FGF23 levels with dietary **(A, B)** and plasma **(C, D)** concentrations of calcium in broilers **(A, C)** and egg-laying hens **(B, D)**. The regression was studied using Bayesian correlation. The number of datasets, Person’s r and Bayes factor are reported below each plot.

**FIGURE 7:**
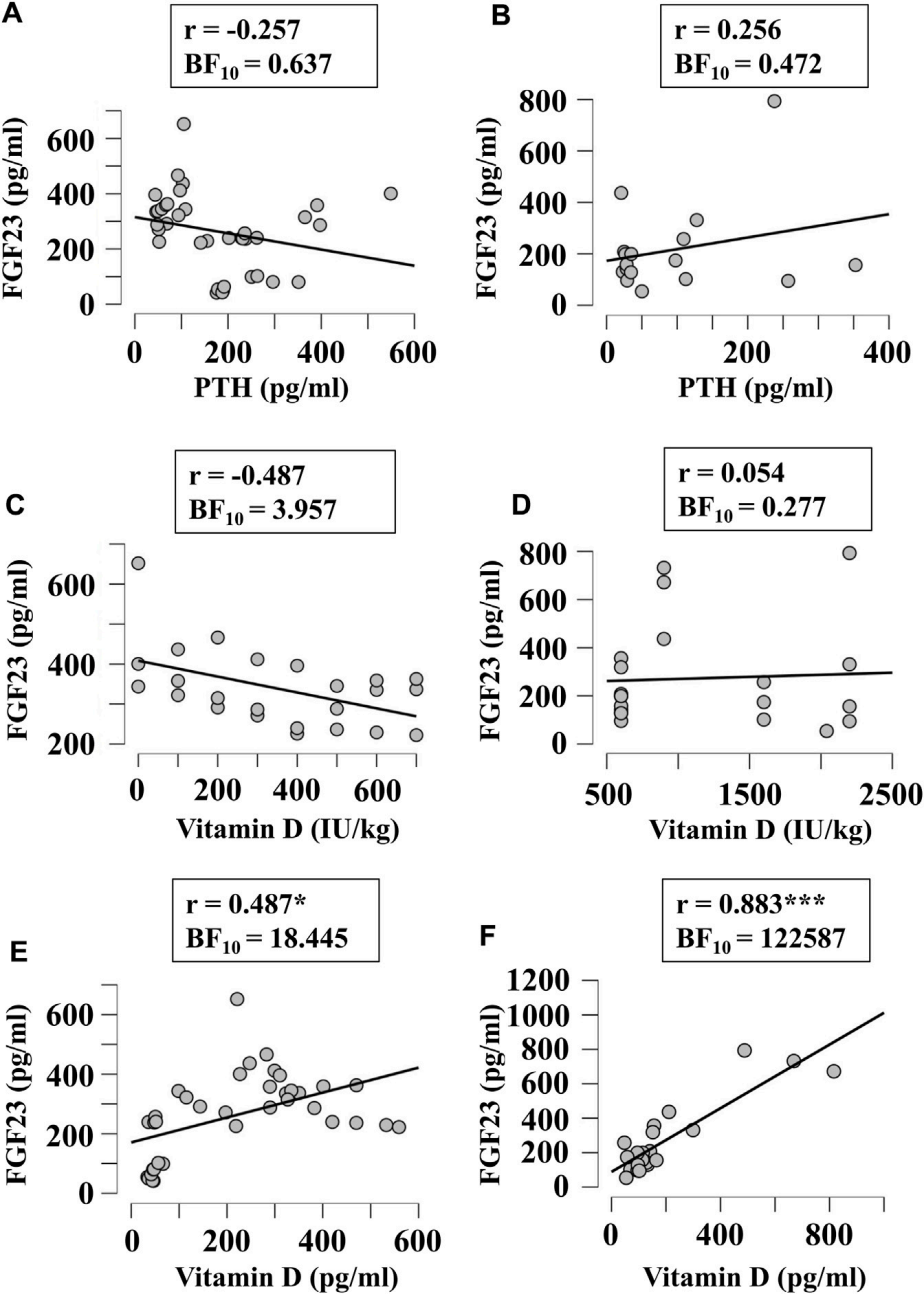
Correlation of blood FGF23 levels with plasma level of PTH **(A, B)**, and dietary **(C, D)** or plasma **(E, F)** levels vitamin D in broilers **(A, C, E)** and egg-laying hens **(B, D, F)**. The regression was studied using Bayesian correlation. The number of datasets, Person’s r and Bayes factor are reported below each plot.

Next, we examined if FGF23 levels correlated with other factors relevant to phosphate and calcium homeostasis, when they were given in diet in variable amounts or measured in blood. Since FGF23 is strongly involved in regulation of phosphate levels, we first examined its correlation with the phosphorous level of diet (Figures 5A,B) and blood (Figures 5C,D) in broilers (Figures 5A,C) and egg-laying hens (Figures 5B,D). There was a moderate evidence (BF_10_ > 3) for positive correlation between plasma phosphorous and FGF23 (Figures 5C,D). To assess the potential validity of this trend, we combined the data from all chicken, which resulted in a significant positive correlation between blood phosphorous and FGF23 (r = 0.45, BF_10_ = 77). Next, we examined if FGF23 levels correlate with dietary (Figures 6A,B) or blood (Figures 6C,D) calcium levels in broilers (Figures 6A,C) and egg-laying hens (Figures 6B,D). Similar to phosphorus, no linear correlation between FGF23 and calcium levels was observed. Finally, we assessed if FGF23 levels correlate to the levels of other hormones involved in calcium and phosphate homeostasis, PTH and vitamin D. Blood levels of PTH and FGF23 did not demonstrate correlation in broilers (Figure 7A) or egg-laying hens (Figure 7B). Dietary vitamin D level demonstrated moderate negative correlation with FGF23 in broilers (Figure 7C), but not in egg-laying hens (Figure 7D). When the datasets from a single study performed with very high dietary vitamin D (Cao et al., 2021) were added to the broilers group, strong negative correlation of vitamin D with blood FGF23 level (BF_10_ = 2.548e+6) was observed, however since this correlation was strongly influenced by a single study, the validity of this association needs to be verified. In contrast, the blood vitamin D levels correlated positively with FGF23 levels in both broilers (Figure 7E) and egg-laying hens (Figure 7F). Thus, from all the parameters of calcium and phosphate homeostasis only vitamin D appeared to have a strong effect on blood levels of FGF23.

## Discussion

In this study, we quantitatively summarized the findings from 12 manuscripts that measured the blood levels of FGF23 in poultry chickens. Broilers and egg-laying hens of seven different breeds were estimated to have similar blood FGF23 level. There was an apparent increase in the blood FGF23 levels with age in both broilers and egg-laying hens, although the analysis was limited by data availability and the timescale to see the effect of age was different for the two groups. Blood FGF23 levels did not correlate with dietary levels of calcium, phosphorous, or vitamin D. While blood FGF23 levels demonstrated a trend to positively correlate with plasma phosphate, no correlation was observed with plasma calcium or PTH levels. Importantly, blood levels of FGF23 and vitamin D correlated strongly and positively in both broilers and egg-laying hens. Our analyses were based on a remarkable number of individual chickens considering the time requirement, complexity and cost of performing animal trials and measurements: we combined the data from 435 broilers from 3 different breeds and 208 egg-laying hens from 4 different breeds varied in age from 7 to 511 days. Thus, we demonstrate that quantitative synthesis of data allows to dramatically increase the power to generate the normative estimates of FGF23 levels in poultry birds and to examine complex relationships between the study parameters.

We estimated the FGF23 levels in broilers and egg-laying hens as remarkably similar, 256 [215, 297] pg/mL in broilers and 256 [178, 339] pg/mL in egg laying hens. In mammals, we have found studies reporting measurements of FGF23 blood levels in humans, mice, rats, dogs, and cats (Table 2). The systematic search and estimation for different animals was not in the scope of the current work; however, Table 2 gives a sense of the ranges of FGF23 levels reported for different species. Of interest, FGF23 levels in humans appear to be the lowest among mammals, at 20–40 pg/mL, followed by estimates in cats of 50–200 pg/mL. Rodents appear to have a higher variability and higher extremes in their FGF23 levels. In birds, blood FGF23 levels were much higher than in humans, but comparable to other mammals. These data suggest that FGF23 levels in birds are not dramatically different from other terrestrial animals.

**TABLE 2:** Comparison of reported blood FGF23 levels in human and several different animals. The reported values are Mean (±sd) or Mean ± SEM, or Median [Min-Max range].

Study/species	Sample size	FGF23 level (pg/mL)
Human studies		
Braithwaite, Jones et al., 2014 (children)	24	28.2 (±12.5)
Burnett, Gunawardene et al. et al., 2006	35	35 (±15)
Smith, Cai et al. 2012	12	26.1 (±6.4)
Vervloet, van Ittersum et al. 2011	10	31 (±5)
Irzyniec, Boryń et al. 2020 (elderly)	11	43.8 (±40.3)
Animal studies		
*Mice*		
Liu, Zhou et al. 2006 (C57BL/6)	15	91.9 ± 7.0
Wang, Buckendahl et al. 2018 (C57BL/6J)	7	426 (±82)
Ryan, Ketha et al. 2013 (C57BL/6)	8	151 ± 11
Fleet, Replogle et al. 2016 (CAST)	6	31.50 ± 65.52
Fleet, Replogle et al. 2016 (C3H)	7	255.37 ± 28.34
Fleet, Replogle et al., 2016 (AKR)	7	213.08 ± 51.62
Wöhrle, Bonny et al. 2011 (Balb/c)	7	62 ± 11
*Rats*		
Aniteli, de Siqueira et al. 2018 (Wistar)	7	968 (573, 2,277)
Liu, Chong et al. 2021 (Male Sprague–Dawley)	10	909.30 (±75.25)
Ferreira, Ferrari et al. 2013 (Male Wistar)	7	286 (±92.2)
Asowata, Olusanya et al. 2021 (male Sprague-Dawley)	9	571.50 ± 66.06
*Dogs*		
Corsini, Dondi et al., 2021 (breed not reported)	12	448.7 [244.8–753]
Miyakawa, Nagatani et al. 2020 (breed not reported)	15	257 [164–563]
*Cats*		
Liao, Chou et al. 2019 (different breeds)	20	121.05 (±71.85)
Paßlack, Schmiedchen et al. 2016 (European Shorthair)	10	101 (±58)

Our findings indicated that blood FGF23 levels are remarkably similar in broilers and egg-laying hens and comparison between the 7 breeds did not show any outlier breed. We could not investigate the effect of sex, because of the poultry industry practices and deficient reporting of sex in studies of young chicken. However, given how similar blood FGF23 is in broilers (mostly male birds) and egg-laying hens suggest that sex is not a strong determinant for FGF23 levels. Similarly, no effect of sex was observed in healthy mice (Tippen et al., 2021) or humans (Stanczyk et al., 2021). FGF23 levels showed significant positive age-dependance in both broilers and egg-laying hens, although on different time scales. In contrast, FGF23 levels were reported to be independent of age in healthy mice (Tippen et al., 2021) and healthy young humans (Mitchell et al., 2017; Stanczyk et al., 2021). It is possible that poultry birds have more significant developmental demands on calcium and phosphate homeostasis, associated with rapid mass gain in broilers and eggshell production needs in hens, which result in a significant FGF23 age dependence compared to mammals.

This study allowed us to examine if blood FGF23 levels correlate with other factors relevant to calcium and phosphate homeostasis in poultry chicken. Plasma calcium and phosphate levels are tightly controlled to ensure the functionality of the essential physiological phenomena these two ions contribute to calcium and phosphorous fluxes in three main organs, intestine, kidneys and bone, are regulated by an interplay between several hormones, the most influential of which are the biologically active form of vitamin D (1,25-dihydroxyvitamin D3), parathyroid hormone (PTH), and FGF23 (Renkema et al., 2008). In meta-analysis, we have not found strong evidence for the correlation of FGF23 with dietary calcium, phosphorus, or vitamin D. Even though we cannot be certain that other dietary factors were similar between different studies, this finding is consistent with those of individual studies (Jiang et al., 2015; Horvat-Gordon et al., 2019; Ren et al., 2020; Cao et al., 2021). When we assessed the correlation between plasma FGF23 and P, only an anecdotal to moderate evidence for such association was found. Although this is somewhat surprising, as FGF23 is a phosphate regulating hormone, this finding is consistent with lack of association between FGF23, and phosphorous reported in several studies in healthy humans (Ferrari et al., 2005; Mitchell et al., 2017). We have similarly found no strong association between FGF23 and plasma calcium or PTH in poultry chicken. In contrast, positive correlation between serum calcium and FGF23 levels was reported in young healthy humans (Mitchell et al., 2017). Importantly, we have found a strong positive correlation between FGF23 levels and plasma vitamin D for both broilers and egg laying hens. In contrast, a number of human studies reported a significant negative correlation of plasma FGF23 and vitamin D (Ferrari et al., 2005; Vervloet et al., 2011; Mitchell et al., 2017). Thus, comparison of the associations between different factors contributing to calcium and phosphate regulation in birds with those in humans indicates that there are potentially unique aspects of calcium and phosphate homeostasis in chickens. However, the differences between such associations in healthy humans and those with diseases affecting kidney, such as chronic kidney disease (Stubbs et al., 2007), suggests that poultry chicken may demonstrate different relationships between factors of calcium and phosphorous homeostasis due to higher physiological demands on these systems for mass gain in broilers and eggshell formation in hens.

We recognize that there are limitations to this work. First, two-third of the studies are from the same research team, which has been focused on chicken’s phosphate metabolism for several years, yet this could be considered as a potential source of bias. We attempted to mitigate this in the subgroup analysis by creating age subgroups that would include datasets from different studies or research teams, if possible. Another limitation was in our inability to examine the effect of sex, which could be an important factor in calcium and phosphate metabolism considering the difference in the physiology and calcium-phosphorous requirements in different sexes. While poultry industry practices make is very difficult to directly assess the effect of sex, striking similarity in FGF23 levels in broilers and egg-laying hens suggest that in chicken sex is unlikely to significantly affect FGF23 levels. Similarly, we were not able to fully investigate the effect of age, since only the datasets on broilers had a good distribution over different ages, while for the egg-laying hens there were large age gaps. This work had also statistical limitations, primarily due to poor reporting of the exact nature of the measure of variance in a number of studies and required the correction, which can potentially affect the precision of the result. Nevertheless, we believe that using RoBMA allowed us to estimate the summary measures with proper prudence and perform the subsequent analysis.

Taken together, our study demonstrates that FGF23 levels in poultry chicken are similar between chicken of different types and breeds, and are within the range reported for other terrestrial animals. Our analysis suggests that further studies are required to understand how physiological demands of mass gain in broilers and eggshell formation in egg-laying hens shape the regulation of their calcium and phosphate homeostasis.

## Data Availability

The data underlying this article will be shared on reasonable request to the corresponding author. Requests to access these datasets should be directed to svetlana.komarova@mcgill.ca.
